# Breast Immunology Network: Toward a Multidisciplinary and Integrated Model for Breast Cancer Care in Italy

**DOI:** 10.3390/cancers17183089

**Published:** 2025-09-22

**Authors:** Andrea Botticelli, Ovidio Brignoli, Francesco Caruso, Giuseppe Curigliano, Vincenzo Di Lauro, Carla Masini, Mario Taffurelli, Giuseppe Viale

**Affiliations:** 1 Medical Oncology Unit B, Policlinico Umberto I, 00161 Rome, Italy; andrea.botticelli@uniroma1.it; 2 Fondazione SIMG, SIMG—Italian Society of General Medicine, 50142 Firenze, Italy; docbri53@gmail.com; 3 Breast Unit, Humanitas Istituto Clinico Catanese, SP54, Contrada Cubba Marletta, 11, 95045 Misterbianco, Italy; francesco.caruso@humanitascatania.it; 4 European Institute of Oncology IRCCS, 20141 Milan, Italy; giuseppe.curigliano@ieo.it; 5 Department of Oncology and Hemato-Oncology, University of Milano, 20133 Milan, Italy; 6 Department of Breast & Thoracic Oncology, Division of Breast Medical Oncology, Istituto Nazionale Tumori IRCCS ‘Fondazione G. Pascale’, 80131 Naples, Italy; 7 Istituto Scientifico Romagnolo per Lo Studio e La Cura Dei Tumori (IRST) IRCCS, 47014 Meldola, Italy; carla.masini@irst.emr.it; 8 Senonetwork Italia, 63822 Florence, Italy; mario.taffurelli@unibo.it; 9 European Institute of Oncology, 20141 Milan, Italy; giuseppe.viale@ieo.it

**Keywords:** breast cancer, immunotherapy, multidisciplinary team, breast immunology network, triple-negative breast cancer, precision medicine, continuity of care, diagnostic-therapeutic pathway, hub and spoke, comprehensive cancer care network, key performance indicators

## Abstract

**Background:** Breast cancer is the most common female cancer in Italy. Despite better survival rates, significant disparities in access to diagnosis, treatment, and follow-up persist across regions. We propose an integrated, multidisciplinary care model—the Breast Immunology Network (BIN)—to address these challenges. **Methods:** The model was developed through a two-phase expert consultation with key opinion leaders and stakeholders, aligned with national and European oncology guidelines. No new patient data were collected; this is a qualitative analysis based on expert consensus and existing literature. The proposed model integrates a Hub-and-Spoke cancer network structure with fully functioning multidisciplinary teams (MDTs), standardized care pathways (PDTA), and digital tools to ensure continuity of care. **Results:** Experts identified critical gaps in Italy’s breast cancer care: limited access to specialized centers, inconsistent adherence to screening programs, and delays in treatment initiation. The proposed BIN model aims to bridge these gaps by enhancing collaboration across all care levels, incorporating immunotherapy where appropriate, and defining key performance indicators (KPIs) for continuous quality evaluation. For example, quantitative targets include achieving ≥65% nationwide mammography screening adherence and ensuring ≥90% of patients are treated in certified Breast Units. **Conclusions:** The Breast Immunology Network offers a strategic framework to improve equity, quality, and timeliness of breast cancer care in Italy. Importantly, unlike existing Hub–Spoke or CCCN models, the BIN formalizes governance tools, harmonized eligibility criteria, and a national registry for immunotherapy. By uniting Breast Units and community services under shared governance, and by integrating innovations such as immunotherapy and telemedicine, the BIN model could significantly improve clinical outcomes and ensure more equitable care for all patients. Its implementation may serve as a reference model for other health systems seeking to optimize oncology pathways through multidisciplinary integration and advanced treatments.

## Simple Summary

Breast cancer is the most common cancer among women in Italy. Although survival has improved thanks to earlier diagnoses and new treatments, many patients still face unequal access to care depending on where they live. This paper proposes the Breast Immunology Network (BIN), a coordinated system that brings together healthcare professionals and centers to ensure more consistent, personalized, and timely care. By reducing delays in diagnosis and treatment (especially for aggressive types like triple-negative breast cancer) and improving follow-up and communication between hospitals and local health services, the network aims to provide high-quality care to all patients, regardless of location. The model emphasizes collaboration, digital tools, and shared standards. Importantly, the BIN model also formalizes innovations not yet uniformly adopted across existing oncology networks—such as a national immunotherapy registry, harmonized eligibility criteria for checkpoint inhibitors, and structured community integration with general practitioners and pharmacists. This approach could serve as a reference for improving cancer care in other settings.

## 1. Introduction

Breast cancer is the most common female cancer in Italy, representing 30% of all diagnoses, with 53,686 new cases in 2024 and an estimated increase of +0.2% per year [1]. Despite a growing incidence, overall survival has improved in recent years (88% at 5 years). Currently, 925,000 women are living with breast cancer in Italy, thanks to earlier and more complex diagnoses, innovative therapies, and personalized care [1].

In Italy, women who are 50–69 years old are guaranteed a mammography every two years, with some regions extending this to women aged 74 years [2]. Annual mammography screening for women aged 45–49 years is also being tested in certain areas. Women who are at high risk typically undergo MRI once a year [2]. Diagnosis of breast cancer is achieved with clinical criteria, including a genomic classification that identifies four molecular subtypes: luminal A, luminal B, HER2-positive, and basal-like [3]. Early diagnosis is crucial to improve breast cancer outcomes. Importantly, national guidelines highlight the importance of early detection to enhance outcomes and reduce treatment invasiveness [4]. Treatment options are chosen based on the diagnosis and include tumor excision, sentinel lymph node removal, radiotherapy, chemotherapy, hormonal therapy, targeted therapy, and immunotherapy [5].

Patients also need continuous and integrated care throughout their treatment, covering all stages from diagnosis to palliative care. A multidisciplinary team (MDT) should be involved in all phases of breast cancer management [6]. An MDT typically includes oncologists, radiologists, nuclear medicine specialists, and surgeons who will discuss personalized treatment options for the individual patient [6]. Additional experts, such as physiotherapists and psychologists, may offer further support as needed [6,7]. The main benefit of an MDT is that it provides consistent, evidence-based, coordinated, and cost-effective care for patients, and the combined knowledge of a medical team leads to better health outcomes than isolated decision-making [6,7].

In Italy, specialized breast cancer centers, known as “Breast Units” or Centri di senologia, focus on prevention, diagnosis, treatment, and follow-up. Established in December 2014, there should be one center for every 250,000 inhabitants, staffed by a team of six specialists and handling at least 150 new cases each year. However, the application of national guidelines varies across regions, leading to inconsistencies in care. Team meetings generally take place weekly, but involvement of general practitioners is often lacking. Each patient undergoes a structured diagnostic and treatment process called a PDTA (diagnostic and therapeutic care pathway), although regional differences exist in protocols and updates [8]. Some Breast Units are certified by Breast Centers Certification programs such as EUSOMA in Europe. EUSOMA certification is a recognition of the best services in terms of organization, diagnosis, and therapy, ensuring that quality controls are followed to meet predetermined standards [9].

Some Breast Units are registered with “Senonetwork”, an Italian association that aims to improve the treatment of breast diseases in Italy by ensuring that facilities meet European standards and offer equal care to all women [10]. Joining this network requires measuring quality through specific metrics and creating a shared database for the collection of information. Regional Oncology Networks (ROR) connect individual Breast Units and facilitate access to diagnosis and treatment, aiming for uniform services and efficient care pathways. The status of ROR varies by region, with some regions lacking such networks while others have had them in place for years. Indeed, a national survey in 2021 documented the presence of diverse ROR models in Italy, including Hub & Spoke models and Comprehensive Cancer Care Networks (CCCN) [11].

The Hub & Spoke model, used in some regions (such as Veneto, Trentino-Alto Adige, Emilia Romagna, Puglia, Calabria, and Sicilia), consists of a series of peripheral centers spread throughout the region (spokes) where most cases are managed, and centers of excellence (hubs) where the most complex cases are handled [12]. A CCCN is overseen by a regional central authority that coordinates the overall programming of the different complementary centers and establishes the organizational, structural, technological, and quality standards for performance and activities. In detail, three operational levels can be identified within such a network:

• First-level centers: Institutions that offer nearby oncology services (e.g., community hospitals, community clinics).
• Second-level centers: District hospitals that, in tandem with third-level centers, carry out diagnosis, treatment, and provide care services for oncology and hemato-oncology patients.
• Third-level centers: Multi-specialist hospitals with highly specialized skills and technological equipment, which also carry out training and research. The ultimate goal of a CCCN is to guarantee person-centered, high-quality care that is uniformly distributed and provides continuity of care between hospitals and the community [13].

Herein, we describe a proposal for a Breast Immunology Network in Italy, a dedicated system designed to provide patient-centered, multidisciplinary, and equitable care that incorporates widespread screening and innovative treatments.

## 2. Materials and Methods

Study design. This was a qualitative, expert-opinion study designed to develop a consensus-based framework for the Breast Immunology Network (BIN). The process followed a two-phase design consisting of individual semi-structured interviews with Key Opinion Leaders (KOLs), followed by a multidisciplinary expert panel discussion. The study was conceived as a qualitative analysis and did not involve patient-level data; therefore, ethics committee approval was not required.

Selection of Key Opinion Leaders (KOLs). KOLs were selected through purposive sampling to ensure both scientific authority and geographic representativeness. Inclusion criteria were: (i) senior leadership roles (e.g., heads of oncology or breast cancer departments, presidents or board members of national scientific societies); (ii) ≥10 years of clinical or academic experience in breast cancer; (iii) active participation in national or regional oncology guideline committees or networks; and (iv) geographic balance across Northern, Central, and Southern Italy. Eleven KOLs meeting these criteria were invited via e-mail with a project briefing and all agreed to participate.

Interviews. Individual semi-structured interviews (n = 11) were conducted virtually (video conference) between September and October 2023. Interviews lasted 30–45 min and followed a structured questionnaire, which explored topics including current challenges in breast cancer care, the role of immunotherapy, MDT functioning, hospital–community integration, and digital health. With consent, interviews were audio-recorded and transcribed verbatim.

Qualitative analysis. Two independent researchers reviewed the transcripts and performed a thematic content analysis. Codes were generated inductively, grouped into categories, and synthesized into thematic domains (e.g., access to Breast Units, molecular diagnostics, integration of immunotherapy, MDT optimization, continuity of care). Discrepancies in coding were resolved by consensus. This process yielded a set of major themes and sub-themes that were used to draft a structured scenario document.

Expert panel. In Phase 2, a virtual multidisciplinary expert panel (2 h) was convened in October 2023. Panelists included the interviewed KOLs and additional stakeholders (hospital administrators, regional health managers, and patient association representatives). A moderator presented the scenario document, which was then discussed and refined by the panel. The discussion was audio-recorded and summarized in a written report. The final outcome was a consensus framework for the BIN model.

**Figure 1:**
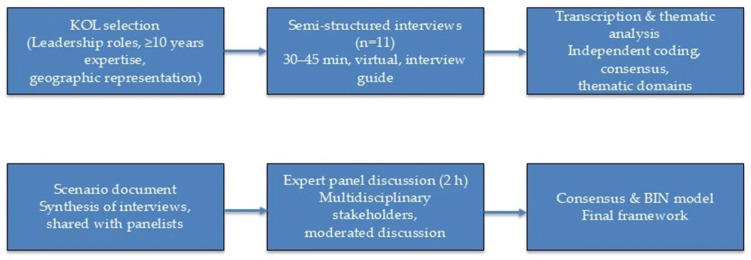
Study process for the development of the Breast Immunology Network (BIN) model.

Transparency and reporting standards. To ensure methodological transparency, the study design and reporting were informed by the Consolidated Criteria for Reporting Qualitative Research (COREQ), a 32-item checklist for interviews and focus groups [14]. The overall process is summarized in Figure 1.

## 3. Results

### 3.1. Current Status of Breast Cancer Management in Italy

Analysis of the experts’ considerations identified several shortcomings in the current system.

#### 3.1.1. Access to Specialist Centers

Specifically, approximately 15–20% of patients are not treated in dedicated breast cancer centers. Currently, reference centers for breast cancer are represented by certified “Breast Units [13].

#### 3.1.2. Role of the Oncology Pharmacist

The role of the hospital pharmacist in breast cancer care needed to be redefined. It was shown that the presence of an oncology-specialized pharmacist increased the safety of drug therapy and the effectiveness of treatment, with positive economic benefits and better acceptance of clinical interventions [15].

#### 3.1.3. Early Diagnosis and Molecular Pathology

Despite enormous progress in molecular diagnostics, the situation remains uneven across regions. Interpretation of advanced results is highly complex and required specialized MDTs, which were only available in some areas [13].

#### 3.1.4. Optimization of Diagnostics

By contrast, bureaucratic, technological, and logistical inadequacies often resulted in incomplete reports, leading to treatment delays with potentially harmful consequences

#### 3.1.5. Neoadjuvant Therapies and Immunotherapy

The importance of early systemic therapy was highlighted, based on early diagnosis and the involvement of the medical oncologist in the MDT. For stage II or III tumors, preoperative (neoadjuvant) systemic therapy provided clinical benefits, including downstaging of the tumor and thus impacting surgical options [16]. Preoperative treatment also helped individualize therapy based on the extent of response. It served as a prognostic marker, identifying women with residual cancer who may warrant additional adjuvant systemic treatment.

Recent developments paved the way for promising new breast cancer therapies, such as immunotherapy [17]. At that time, immunotherapy was approved in the neoadjuvant setting for PD-L1–positive early triple-negative breast cancer, as shown in the phase 3 KEYNOTE-522 trial [18]. In that trial, the addition of pembrolizumab to platinum-containing neoadjuvant chemotherapy showed a significant improvement in 5-year overall survival (86.6% vs. 81.7% in the placebo arm) among patients with early-stage triple-negative breast cancer [18]. Immune checkpoint inhibitors (ICIs)—antibodies that enhance the anti-cancer immune response by blocking inhibitory pathways in immune cells—have also yielded promising results in breast cancer [19]. Studies of the immune system’s role in breast cancer reveal that a subset of aggressive tumors, such as triple-negative cancers with high tumor-infiltrating lymphocytes, have substantial immune cell presence in the tumor microenvironment [20]. A recent meta-analysis of nine randomized trials (5114 patients) confirmed that adding ICIs to neoadjuvant chemotherapy significantly increases pathological complete response rates and improves event-free survival in early-stage triple-negative breast cancer [21].

#### 3.1.6. Continuity of Care Between HOSPITAL and Community

It was evident that hospital facilities played a major role in oncology care, but focusing solely on hospital-centered treatment was outdated.

#### 3.1.7. Screening and Prevention Programs

In 2023, adherence to mammography screening programs was on average 49% nationally, with a stark difference between Northern and Southern Italy (approximately 62% vs. 31%, respectively) [1,22]. Importantly, closing this gap was a priority and was monitored through explicit KPIs on screening uptake (see Table 1).

### 3.2. Objectives and Purpose of the New Model

Based on the experts’ considerations and taking into account national (in particular the National Cancer Plan 2023–2027) and European guidelines for breast cancer care, we defined the following objectives for the proposed model:

• Establish a dedicated cancer network, termed the “Breast Immunology Network.”
• Recognize the central role of the patient in the care pathway.
• Enhance personalized medicine and precision therapies.
• Provide equitable and improved access to prevention, screening, diagnosis, and treatment, with a focus on at-risk groups.
• Improve early detection and treatment, especially for aggressive cancer types with limited treatment choices (e.g., triple-negative breast cancer), based on criteria of effectiveness, efficiency, and appropriateness.
• Foster access to new treatments, including experimental ones, with diagnostic-therapeutic pathways (PDTA) that facilitate early use of immunotherapies.
• Ensure uniformity and proximity of care throughout all regions.
• Provide continuity of care and assistance between the hospital and the community.
• Include general practitioners and pharmacists as active participants in the network.
• Share guidelines and scientific knowledge among healthcare providers and facilities.
• Encourage teamwork and information-sharing with organizations, universities, patient associations, and other groups (including those not formally part of the network).
• Ensure digitization of regional and national processes to allow seamless exchange of information and health records.
• Encourage patient enrollment in clinical trials.

### 3.3. Structural and Organizational Structure of the New Model

In terms of organizational setup, the model includes a network of different structures in a mixed Hub-and-CCCN configuration [23]. In this context, each center needs to have adequate data storage and telemedicine software, allowing patients to be monitored at home or at smaller local centers while still receiving specialist consultation from the main hubs. The “hubs” (central reference centers) are selected based on criteria such as organizational capacity, available technologies, level of activity, and experience. They can handle more complex tasks (e.g., scientific research, advanced training, clinical trials, and molecular diagnostics), while the peripheral centers ensure widespread, capillary care, thereby optimizing each step of the diagnostic-therapeutic-assistance pathway. The distribution of tasks among these centers is detailed in Table 2.

The Spoke centers provide on-site support—known as “proximity oncology”—to manage patients who are receiving treatment as well as those in follow-up who are not currently being treated. Such support covers both home care and digital services (e.g., telemedicine). The Hub centers should also develop treatment plans for anti-cancer drugs that are subject to special registries by the Italian Medicines Agency (AIFA), and establish, share, and update regional PDTAs. All centers in the network should satisfy top European standards (for example, be EUSOMA-certified) and be part of the Senonet work. Ultimate oversight of quality-related processes should be managed by external organizations via certification and by the regional Department of Health.

### 3.4. Definition and Functions of the MDT

Each center should have an MDT, consisting at minimum of a breast surgeon, radiologist, radiation oncologist, medical oncologist, pathologist, and nurses with experience in breast oncology. The team should also include or have access to other professionals with relevant expertise, such as a plastic surgeon, geneticist, pharmacist, nuclear medicine specialist, physiatrist (rehabilitation specialist), dietician, psychologist, and experts in palliative care/pain therapy.

Each clinical case should be reviewed by the MDT in a dedicated meeting for both pre- and post-operative assessment to propose the best diagnostic-therapeutic approach and address any clinical issues requiring re-evaluation. Meetings should be held weekly and should conclude with the creation of a digital report that is archived and made available to other centers in the network in order to increase overall expertise. Each patient should be assigned to a “case manager”, who will follow the patient throughout treatment and follow-up.

In this context, each center needs to have adequate data storage and telemedicine software, allowing patients to be monitored at home or at smaller local centers while still receiving specialist consultation from the main hubs [24]. The procedures for collecting, storing, and disseminating data should follow guidelines shared by all centers and be uploaded into a shared database (e.g., a unified Electronic Medical Record system), allowing faster access to information and better patient monitoring.

The network should work closely with voluntary associations, as well as community nurses, general practitioners, and local pharmacists. General practitioners, whose current role in cancer patient management is marginal, should assume a more central role in screening, treatment, and follow-up and be an integral part of the MDT [25]. Through refresher courses, general practitioners should be updated on new diagnostic and treatment options and on the establishment of a breast network in the area, so that they can effectively support patients in need and quickly address their questions.

To this end, it would be useful to facilitate communication and collaboration with the MDT and the case manager—also via remote tools—to ensure complete and effective assistance, especially in the essential steps of the PDTA. The local pharmacist also has a role, not only because pharmacies are widely present throughout communities, but also in active patient management through monitoring of at-home therapies and reporting of adverse effects. Each Breast Unit must have a dedicated surgical team and a specific PDTA for patients at high risk of hereditary breast cancer, as well as a PDTA specific to triple-negative breast cancer. Patients with advanced disease should have access to palliative care and integrated home care services.

### 3.5. Definition of the PDTA

The PDTA (diagnostic and therapeutic care pathway) should be defined by an MDT and updated and/or integrated periodically, based on new evidence, to improve survival and quality of life for patients. In particular, the PDTA should include the following recommendations for women with breast cancer:

• Primary and secondary prevention, as well as tertiary prevention (follow-up) modalities.
• Criteria for entry into the PDTA.
• Timing and modes of diagnosis, staging, and treatment.
• MDT activities (points in the care pathway where case discussion is required).
• Access to neoadjuvant and adjuvant therapies.

Specific phases of care to be carried out according to pre-established timelines, especially for early screening access; imaging, radiological and interventional diagnostic examinations; and integrated histological and genetic diagnosis.

Biopsy material should be sent to laboratories that specialize in molecular diagnostics and genomic profiling. In this regard, the availability of a Molecular Tumor Board is warranted, which can interpret the results of complex molecular tests performed on tumor tissue or liquid biopsies that identify various alterations (genomic, epigenomic, transcriptomic, proteomic, metabolomic), thereby allowing prediction of response to targeted therapies [26]. The role of this committee should be advisory, and the final choice of therapy will be made by the chair of the MDT.

In case of a positive (malignant) diagnosis, the patient will be taken into care and the most appropriate PDTA will be established. The case manager will be the patient’s point of reference throughout their journey. Depending on the clinical situation, the process will be referred to either Medical Oncology or Breast Surgery. In fact, pre-surgical (neoadjuvant) oncology therapy is recommended in clinical practice guidelines in order to reduce the size of the tumor before surgery and to assess the residual tumor at the end of medical therapy.

If surgery is performed first, an anatomopathological analysis of the tumor tissue should be carried out, and after the report is received a second MDT meeting should be held to reassess the case. In this context, stratification of patients for adjuvant therapy is crucial. The importance of timely reporting and regular team meetings to proceed to the next step is emphasized. This should be followed by a second consultation with the patient, in which the final pathology results and the recommendations for the next phase of treatment (which may include radiotherapy and/or chemotherapy and/or immunotherapy) are discussed in detail, including explanations of administration methods, doses, duration, and potential side effects. Subsequently, patients will be scheduled for regular follow-up.

### 3.6. Quality of the Network and Evaluation Through Key Performance Indicators

A requirement of modern healthcare systems is to ensure a high level of quality in terms of accessibility, appropriateness, equity, effectiveness, and efficiency. The network should be managed through a policy of clinical governance, formalized in a document that employs the following tools:

• Continuous training and updating of health professionals.
• Implementation of information systems (e.g., electronic medical records).
• Implementation of PDTAs based on evidence-based medicine.
• Clinical risk management.
• Clinical audits.

Clearly defined work structures, processes, roles, and methods, with activity tracked and evaluated using key performance indicators (KPIs) to oversee processes and apply corrective measures [27].

To ensure high-quality patient care, performance must be continuously monitored. Table 1 summarizes the core KPIs adopted to evaluate the network.

In the network’s policy document, defining these KPIs and the target values for their achievement is crucial to assess objectives, and this may be tied to resource distribution.

Additional specific indicators can be used for more detailed evaluations. For example, key KPIs for screening programs include:

• Percentage of patients who underwent a histologic assessment after a positive screening test.
• Positive predictive value of screening (the percentage of lesions found to be malignant among those that tested positive).
• Recall rate (the proportion of screened women who are called back for further diagnostic investigations).
• Detection rate (the number of malignant lesions diagnosed per 1000 women screened).
• For MDT performance evaluation:
• Percentage of patients who were discussed by an MDT before receiving any therapy.
• Percentage of patients whose treatment was overseen by an MDT at each stage of the disease.
• Percentage of patients for whom documentation regarding the intended treatment plan is sent to the general practitioner within a specified time frame (either before an MDT meeting or at any other time during the course of treatment).

For assessment of diagnosis and treatment pathways, the following indicators may be used:

• Volume of cases (number of new breast cancer cases managed).
• Percentage of patients who received treatment (out of those diagnosed or discussed by the MDT).
• Percentage of patients with invasive cancer who required only a single surgical operation.
• Percentage of new cases in which surgical treatment was initiated within 18 days after diagnosis.
• Percentage of patients with invasive carcinoma who received radiotherapy.
• Percentage of HER2-positive patients treated with chemotherapy plus trastuzumab and pertuzumab.
• Percentage of high-risk early TNBC patients treated with neoadjuvant pembrolizumab + chemotherapy.
• Percentage of patients who migrated to other centers for treatment.

In the context of innovative therapies and outcomes, additional indicators such as overall survival, progression-free survival, and quality of life can be assessed. Moreover, new indicators should be introduced in light of emerging evidence.

## 4. Discussion

In this paper, we proposed the Breast Immunology Network as a novel, integrated model to enhance breast cancer care across Italy. This network builds on existing structures, such as certified Breast Units and Regional Oncology Networks (ROR), but introduces new elements to address persistent gaps. Notably, it is designed to address regional disparities in access and outcomes. By contrast, breast cancer care guidelines currently vary across Italian regions, leading to inconsistent practices. Crucial players such as general practitioners (GPs) are often not integrated into oncology care teams. Specifically, as is highlighted in the Results, screening uptake remains heterogeneous across regions, requiring targeted actions to improve participation in underserved areas. Actively engaging primary care physicians and community pharmacists in screening outreach is crucial. For example, personalized reminders, mobile apps, or social media campaigns could help boost participation in underserved regions. These discrepancies underscore the need for a coordinated national network to ensure equitable, standardized care regardless of a patient’s location. Establishing new certified Breast Units in underserved regions and engaging primary care physicians more actively in patient referral are critical to improving access to specialized care. By uniting hospitals and community services under a common framework, the proposed model aims to guarantee continuity of care from diagnosis through survivorship, thereby bridging the traditional divide between tertiary centers and local healthcare providers. Given the chronic nature of breast cancer, greater emphasis should be placed on community-based services and assigning a case manager to each patient to ensure consistent follow-up and support.

Our proposal aligns with and extends previous models and evidence in oncology care. The hub-and-spoke structure embedded in the Breast Immunology Network is consonant with the Comprehensive Cancer Care Network (CCCN) approach already adopted in some Italian regions, which seeks to centralize complex care in high-tier centers (hubs) while maintaining widespread local access (spokes). We also emphasize the role of multidisciplinary teams (MDTs) at every level of the network, reflecting the well-established benefits of MDT involvement: coordinated, personalized treatment planning by a team of specialists has been shown to improve patient outcomes compared to isolated decision-making. Unlike the existing Breast Unit system—which, despite national guidelines, still shows variability and patchy implementation across regions—the Breast Immunology Network would enforce shared protocols and uniform quality standards across all centers. Each participating center would be expected to meet top European quality criteria (e.g., EUSOMA certification) and participate in unified data collection, ensuring that best practices are uniformly applied. By integrating these standards with a dedicated focus on immuno-oncology, the network represents an evolution of Italy’s oncology infrastructure toward greater consistency, quality oversight, and innovation in care delivery.

Importantly, a key innovation of the proposed network is its systematic incorporation of immunotherapy and precision medicine into early breast cancer management. Recent clinical breakthroughs have highlighted the value of immunotherapeutics in breast cancer—for instance, adding the immune checkpoint inhibitor pembrolizumab to neoadjuvant chemotherapy significantly improved 5-year overall survival in patients with high-risk early triple-negative breast cancer. However, access to such cutting-edge treatments and molecular diagnostics can be uneven, and their integration into routine practice is not yet universal. Our network model proactively addresses this by including immuno-oncology expertise within MDTs and by establishing pathways that fast-track eligible patients to novel therapies. In doing so, it seeks to democratize access to innovation, ensuring that advancements like ICIs, genomic profiling, or other targeted treatments are available to patients irrespective of region.

Building on recent international recommendations, including the ESMO Clinical Practice Guidelines for Breast Cancer (2024) [28] and the ASCO Guideline Rapid Recommendation Update on immunotherapy for high-risk, early-stage triple-negative breast cancer (2022) [29], the BIN framework goes beyond guideline statements by explicitly addressing their implementation. In particular, the model introduces harmonized eligibility criteria for the use of ICIs across Italian regions, the establishment of a centralized national registry to monitor indications, toxicity, and outcomes, and the integration of sustainability assessments into regional planning. These features provide an operational dimension to guideline recommendations, ensuring that innovations such as chemo-immunotherapy for early TNBC are equitably and safely delivered across the country. Unlike existing Hub–Spoke or CCCN models, which mainly define structural organization, the BIN model formalizes governance tools (registry, shared eligibility, equity measures) required for the systematic adoption of immunotherapy into daily clinical practice.

Furthermore, the Breast Immunology Network leverages digital health solutions to support these clinical innovations. A robust informatics infrastructure—including interoperable electronic health records and telemedicine platforms—will connect hub centers with community healthcare providers in real time. Importantly, this integration will support remote consultations, shared decision-making, and continuous patient monitoring at home. Such tools are especially crucial given the chronic nature of cancer care and the need for long-term follow-up.

The BIN model explicitly integrates community care into oncology pathways. General practitioners lead prevention, survivorship, and timely referral; pharmacists support at-home therapy monitoring and adverse-event reporting; and telemedicine connects spoke centers with hubs, enabling remote MDT participation and second opinions. Importantly, a case manager coordinates transitions across settings, ensuring continuity and adherence.

Overall, these innovations—from immunotherapy integration to digital connectivity and community engagement—differentiate the proposed Breast Immunology Network from existing care models and position it at the forefront of modern oncologic care.

A distinctive feature of the BIN model is the systematic integration of immunotherapy into the early management of breast cancer. In particular, immune checkpoint inhibitors (ICIs) are considered in stage II–III triple-negative breast cancer (TNBC) with PD-L1 positivity, in line with the KEYNOTE-522 trial. Their inclusion in the BIN pathway requires harmonized eligibility criteria across regions, economic sustainability assessments, and the establishment of a centralized national registry to monitor indications, toxicity, and outcomes. This registry would serve both clinical governance and pharmacovigilance purposes, ensuring safe and equitable use of ICIs nationwide.

To guarantee equity of access, the BIN model explicitly addresses the role of spoke centers in peripheral or resource-limited areas. All spoke centers will be linked to hub centers via structured telemedicine platforms. This will allow remote participation in MDT meetings and second opinions for complex cases. Standardized referral pathways and digital protocols will ensure that patients managed in spoke settings receive the same quality of care as those treated in hubs. Additional resources and continuous training programs will be allocated to underserved regions to mitigate disparities in screening coverage, access to molecular diagnostics, and availability of innovative therapies.

Recent national data confirm substantial inequalities in breast cancer prevention across Italy: in 2022, adherence to organized mammography screening programs was approximately 62% in Northern regions compared with only 31% in the South [30]. These differences exemplify the persistent geographic divide that directly impacts early detection and survival outcomes.

The BIN model explicitly addresses these gaps by providing structured telemedicine platforms, standardized referral protocols, and continuous training for healthcare professionals in underserved regions. Additional resources are allocated to peripheral centers to enhance diagnostic capacity and to ensure that patients treated in spoke settings receive the same quality of care as those in hubs.

Despite its promise, the implementation of this network is not without challenges and limitations. Italy’s healthcare system is regionally organized, and substantial heterogeneity in resources and outcomes must be considered. Regions that lack established oncology networks or have historically lower healthcare performance (e.g., lower screening rates or fewer certified Breast Units) may face greater hurdles in adopting the model. Ensuring uniform availability of advanced diagnostics and treatments is a significant concern. For example, some areas may currently lack the laboratory infrastructure for molecular testing or the clinical expertise to administer novel immunotherapies. A phased approach might be necessary, with initial pilot programs in well-prepared regions to demonstrate feasibility and effectiveness. Moreover, standardizing care pathways across diverse institutions will require strong governance and consensus-building. Resistance to change, whether due to administrative inertia or professional autonomy, could impede the harmonization of protocols. The network must therefore incorporate clear incentives and support for compliance, as well as training programs to bring all centers to a common level of readiness.

Another practical limitation is the potential for bureaucratic and logistical obstacles. The current system already suffers from delays in diagnosis and treatment initiation, partly due to bureaucratic bottlenecks and fragmented care processes. Without careful planning, a more complex network could unintentionally add new layers of coordination. These additional steps may further slow down care. To counter this, our model emphasizes streamlining workflows—for instance, by digitizing referrals, simplifying reporting procedures, and using case managers to help navigate patients through the system. Finally, sustainable funding and political will are critical: establishing new digital platforms, expanding MDTs, and maintaining quality oversight (such as independent audits using KPIs) will require dedicated investment. Persistent regional disparities remain a key challenge for implementation and scale-up of the BIN model. Acknowledging these challenges is important, as it allows for proactive strategies to mitigate risks—for example, tailoring implementation plans to each region’s context and establishing an adaptive feedback mechanism as the network rolls out.

An additional limitation to acknowledge is the potential perception that the BIN model merely replicates existing organizational models, such as Hub–Spoke networks or CCCNs. However, the BIN model should not be regarded as a standard already in place and subsequently neglected. Rather, it represents an innovative proposal that explicitly integrates recent advances in immuno-oncology and digital health into the structure of breast cancer care. By incorporating systematic criteria for the use of immunotherapy, a national registry to monitor outcomes, and digital platforms to connect hub and spoke centers, the BIN model extends beyond traditional models. This innovative dimension justifies its development and discussion, as these elements are not yet uniformly implemented in current oncologic networks.

To realize the full potential of the Breast Immunology Network, we outline several key actions and future directions that emerge from this analysis:

• Pilot implementation and policy support. Initiate pilot programs in select regions to evaluate the feasibility and effectiveness of the network on a smaller scale. Insights from these pilots can inform refinements to the model. Concurrently, develop national policies or guidelines that formally integrate this model into Italy’s oncology care framework, including provisions for incorporating immunotherapies and precision medicine into standard practice.
• Strengthening multidisciplinary collaboration. Expand and reinforce the role of MDTs by including all relevant specialists (surgeons, medical and radiation oncologists, radiologists, pathologists, etc.) and by formally integrating general practitioners and oncology pharmacists into the care team. This will ensure that every patient’s case is reviewed from multiple expert perspectives and that care bridges hospital and community settings, improving follow-up and treatment adherence. Regular cross-disciplinary training and joint case discussions (including via teleconsultation) should be encouraged to maintain a high level of expertise and cohesion across the network.
• Optimizing diagnostic–therapeutic pathways. Standardize and streamline the patient pathway from screening to diagnosis to therapy. This entails ensuring uniform access to advanced diagnostic tools (such as molecular subtyping and genetic testing) across all regions, so that early and precise diagnosis is not limited to certain centers. It also involves reducing delays by removing bureaucratic hurdles—for instance, implementing fast-track referral systems and electronic reporting to avoid lost time between detection and treatment initiation. By refining these pathways, the network can shorten time-to-treatment and improve outcomes, especially for aggressive cases where time is critical.
• Enhancing digital infrastructure and data sharing. Invest in a robust digital infrastructure that connects all nodes of the network. A shared electronic health record system should enable real-time sharing of test results, treatment plans, and follow-up information among hospitals and local clinics. Telemedicine platforms are also vital for remote patient monitoring and consultations, ensuring that patients in remote or under-resourced areas receive specialist input without unnecessary travel. Such digital tools will facilitate continuity of care and allow for centralized data collection to evaluate performance metrics.
• Continuous monitoring and quality improvement. Define clear KPIs—for example, screening uptake, time from diagnosis to treatment, adherence to guidelines, patient survival, and quality-of-life outcomes—and monitor these across the network. An independent body or quality assurance program could audit these metrics periodically. Regular feedback based on KPI data will help identify areas for improvement and drive iterative enhancements to the network’s protocols. This continuous evaluation mechanism is essential to maintain high standards and to demonstrate the network’s value to stakeholders and policymakers.

By pursuing these steps, the Breast Immunology Network can be more effectively implemented and scaled up. Over time, such efforts should also be accompanied by further research—for instance, studies to measure patient outcomes and satisfaction under the network model vs. the prior standard of care, or analyses of cost-effectiveness and healthcare utilization within the network. Continued policy support, including sustained funding and possibly legislative backing, will be needed to embed the network into the national health system. In summary, thorough planning, resource allocation, and stakeholder engagement are critical to overcoming implementation barriers. With these in place, the proposed network holds the potential to transform breast cancer care in Italy, making it more patient-centered, equitable, and attuned to the latest scientific advancements.

## 5. Conclusions

The Breast Immunology Network (BIN) represents an innovative evolution of existing oncology frameworks by systematically integrating immunotherapy, precision diagnostics, digital health, and community engagement under shared governance.

It ensures tangible benefits for patients, including more equitable access across regions, earlier and more accurate diagnoses, timely initiation of therapies, and stronger continuity of care throughout the entire pathway—particularly for high-risk subgroups such as early triple-negative breast cancer.

Call to action. Pilot programs and national policy support are needed to test feasibility, refine KPIs and eligibility criteria, and enable sustainable scale-up of the BIN as a model for integrated, equitable cancer care.

## Figures and Tables

**Table 1:** Key performance indicators (KPIs) to evaluate the Breast Immunology Network.

Area	KPI	Target
Screening and Early Diagnosis	% of adherence to mammographic screening	≥65%
	% of molecular biopsies performed in accredited centers	≥80%
Access to Care	% of patients treated in certified Breast Units	≥90%
Multidisciplinarity	% of patients reviewed by an MDT before treatment	≥95%
Innovative Therapies	% of TNBC patients treated with neoadjuvant immunotherapy	≥80%
Clinical Outcomes	5-year survival rate (TNBC)	≥86%
	% reduction in diagnosis-to-treatment time	−20%

**Table 2:** Distribution of competencies among Hub, Spoke, and Comprehensive Cancer Centers.

Structure	Main Functions
Hub (Reference Center)	Advanced diagnostics; clinical research; precision therapy; specialist training; coordination of clinical trials.
Spoke (Territorial Center)	Basic diagnosis; “proximity” oncology (local outpatient treatment); follow-up; delivery of standard therapies (e.g., adjuvant chemotherapy).
Comprehensive Cancer Center (CCCN)	Regional coordination; standardization of care; PDTA management; specialist training.

## Data Availability

The original contributions presented in this study are included in the article. Further inquiries can be directed to the corresponding author.

## Abbreviations

The following abbreviations are used in this manuscript:

CCCN	Comprehensive Cancer Care Network
CT	Chemotherapy
EUSOMA	European Society of Breast Cancer Specialists
HER2	Human Epidermal Growth Factor Receptor 2
ICI	Immune Checkpoint Inhibitor
KOL	Key Opinion Leader
KPI	Key Performance Indicator
MDT	Multidisciplinary Team
MRI	Magnetic Resonance Imaging
PD-L1	Programmed Death-Ligand 1
PDTA	Diagnostic and Therapeutic Care Pathway
ROR	Regional Oncology Network
TNBC	Triple-Negative Breast Cancer
